# Mobile Responsive App—A Useful Additional Tool for Data Collection in the German Pregnancy Register Rhekiss?

**DOI:** 10.3389/fmed.2021.773836

**Published:** 2021-12-17

**Authors:** Jutta G. Richter, Anja Weiß, Christina Bungartz, Rebecca Fischer-Betz, Angela Zink, Matthias Schneider, Anja Strangfeld

**Affiliations:** ^1^Policlinic for Rheumatology and Hiller Research Unit for Rheumatology, Medical Faculty, Heinrich-Heine-University Duesseldorf, University Clinic, Düsseldorf, Germany; ^2^Epidemiology Unit, German Rheumatism Research Center (DRFZ), Berlin, Germany

**Keywords:** digital, app, pregnancy, register, mobile, mobile health application

## Abstract

**Background:** The German pregnancy register Rhekiss is designed as a nationwide, web-based longitudinal observational cohort established in 2015. The register follows women with inflammatory rheumatic disease prospectively from child wish or early pregnancy until 2 years post-partum. Information on clinical and laboratory parameters, drug treatment, and (adverse) pregnancy outcomes are documented in pre-specified intervals. Physicians and patients report data for the same time periods *via* separated accounts and forms into a web-based application (app). As data entry on mobile devices might improve response rates of patients, a responsive app as a further convenient documentation option was developed.

**Methods:** The Rhekiss-app is available for self-reported data retrieval since August 2017 from the App stores. For the current analysis, Rhekiss register data were used from the start of the register until 30 September 2020. The analyses were performed for forms containing information on devices. Outcome parameters were compared for mobile and desktop users for the quantity and quality of filled forms.

**Results:** In total, 5,048 forms were received and submitted by 966 patients. About 57% of forms were sent from mobile devices with the highest numbers in patients with child wishes (63%). Users of mobile devices were slightly younger and often had less high-education level (62 vs. 79%) compared with desktop users. The proportion of forms submitted *via* mobile devices increased steadily from 48% in the fourth quarter of 2018 to 64% in the third quarter of 2020. The proportion of forms received before and after the Rhekiss-app implementation increased with the highest increase of 12% for forms filled at time point 12 months post-partum. Mobile users submitted significantly more forms than desktop users (2.9 vs. 2.1), data sent *via* desktops were more often complete (88 vs. 86%).

**Conclusion:** The responsive app is a valuable additional tool for data collection and is well-accepted by patients as indicated by its increasing use in Rhekiss. Apart from desktop/browser developments, the technological adoptions within observational cohorts and registries should take smartphone requirements and developments into account, especially when patient-reported data in young, mobile patients are collected, bearing in mind that data quality could be compromised and concepts for improving data quality should be implemented.

## Introduction

Advances in family planning and pregnancy management enable more successful pregnancies in women with inflammatory rheumatic diseases (IRD), which are, in general, associated with an increased incidence of complications and adverse pregnancy outcomes (1). Recommendations on the management of family planning and health issues of women have been published (2). Effective pregnancy planning, contraception guidance, and firm treat-to-target goals may decrease the risk for maternal-foetal morbidity and mortality (3, 4). More standardised data on the outcomes of pregnancies and the potential influence of drug exposures in various IRDs are warranted to further optimise management recommendations and continuously evaluate the safety of medications. Thus, registers and the European Network of Pregnancy registers in rheumatology (EuNeP) have been implemented (5, 6).

Information technology (IT) applications (apps) guaranteeing privacy and confidentiality for all stakeholders—most importantly not only at the point of care—seem to be a prerequisite for a successful and sustainable modern register. Digital apps including apps that offer valuable perspectives for modern health care systems and are recommended for health system strengthening by the WHO (7). They may improve and facilitate health services research and reduce barriers to better health for mothers with IRD and their children (8–10). In addition, the increasing use of mobile devices, especially among young adults, and the ability to access data *via* smartphone apps have reshaped data collection procedures (11).

In the nationwide, prospective, observational German pregnancy register “Rhekiss” rheumatologists and patients report data into a web-based app, e.g., on personal computers, laptops, and notebooks (6). This app was not responsive, a prerequisite for self-reported data entry of patients on mobile devices. Thus, the responsive Rhekiss-app for mobile devices was developed as a convenient means for patients to facilitate data entry on a larger number of devices and/or to increase the response rate of patients. We evaluated the quantity and quality of Rhekiss-app data compared with data collected directly through the web app to analyse the suitability of the Rhekiss-app as a tool for the collection of patient-reported data in an online register.

## Materials and Methods

The German pregnancy register Rhekiss is a collaboration among the German Rheumatism Research Centre (DRFZ) in Berlin, the Rheumazentrum Rhein-Ruhr e.V, and the Policlinic for Rheumatology and Hiller Research Unit for Rheumatology at the University Clinic in Duesseldorf, Germany. The register is designed as a nationwide longitudinal observational register and was established in 2015. The website is accessible on https://rhekiss.de and holds register-content related and technical “frequently asked question” information for participating physicians and patients. Content and technical related support are available during business hours *via* phone or email.

The register follows women with IRD prospectively in pre-specified intervals. Patients are included through their rheumatologists. The eligibility criteria of patients comprise of: (1) one of the following IRDs diagnosed by a rheumatologist: rheumatoid arthritis, psoriatic arthritis, spondyloarthritis, juvenile idiopathic arthritis, systemic lupus erythematosus, other connective tissue diseases, vasculitis, and autoinflammatory syndrome; (2) age above 18 years; (3) inclusion with child wish or during pregnancy until 20th week of gestation; and (4) a signed General Data Protection Regulation (GDPR) conform informed consent.

**Figure 1:**
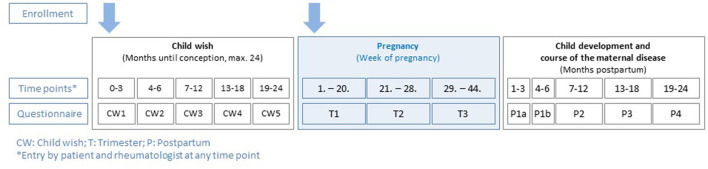
Rhekiss assessment schedule from the three modules (child wish, pregnancy, and post-partum).

Patients are documented in the register in so called “modules” (module A: child wish, module B: pregnancy, and module C: post-partum), scheduled assessments depicting the periodic intervals of data acquisition of patients and physicians are shown in Figure 1. At each assessment, both patients and their rheumatologists report data. During the observations, sociodemographic data, course of the maternal disease (e.g., disease activity and flares), comorbidities, drug treatment, information on clinical and laboratory parameters, development of foetus, respectively, child, complications, and validated patient-reported outcome measurements (PROMs) are assessed.

The technical implementation of the register was carried out in collaboration with a German IT company and a German survey tool manufacturer (Serrala Cloud Solutions GmbH and Tivian XI GmbH). The database of the register was hosted at the University Clinic in Duesseldorf until January 2021 and then moved to a public provider in Germany who is certified to the internationally accepted norm ISO/IEC 20000-1:2011 and ISO/IEC 27001:2013. The study centre is located at the DRFZ in Berlin. The responsive Rhekiss-app was developed in 2017 and is currently available in German only. It can be downloaded for patient-reported data entry from the App stores (Google Play and Apple App Store) free of charge. The app uses common data entry forms for data documentation. Rhekiss does not provide (mobile) devices for register use. A “bring your own device” (BYOD) concept is followed for documentation by patients. Physicians usually use the IT infrastructure of their professional work environment.

Data entry is performed online only by accessing the web-based system in a browser or *via* the Rhekiss-app *via* personalised logins. Forms for data entry have pre-given, varying validity periods that define when reports should be submitted and are automatically submitted with the information entered at this certain point. This applies to the patient and physician system. The approach guarantees that any data entered but not yet sent are delivered to the database automatically. Thus, data loss is kept to a minimum.

The rheumatologists register the patients using personal data and a valid email address of a patient. Pseudonymisation is performed immediately. The patient receives an automatically generated email with a link from the Rhekiss app, which enables independent login to the patient account and the provision of disease specific forms.

The patients activate their accounts *via* a link sent to the email address provided by each patient to the rheumatologist for enrolment in the register. Depending on the active module at inclusion, the patient receives a set of data entry forms (such as, PROs and other data) that need to be completed *via* browser access on personal computers, laptops, and notebooks, or in the corresponding Rhekiss-app. As the observation progresses, the patients receive a one-time email informing them that new questionnaires are ready to be filled out by them in the system. No further reminders are sent within a module. Corrections or the addition of data of patients are not included in the monitoring system that was established for the physician part of the system, as seen below.

For each patient and according to the module, the physician receives a set of web-based data entry forms. Some information (e.g., obstetric history) can be entered at any time during the respective module, but trimester-specific information (such as, medication and disease activity) can only be entered and sent to the database up to a pre-defined date which is pre-calculated based on the reported conception date.

A monitoring system for Rhekiss has been established enabling the Rhekiss analyses team for data protection-compliant enquiries to the rheumatologists and thus correction of data and/or adding missing information or details. Printouts of data for the files of physicians are not provided.

During data entries, unintentional non-response is minimised by some error prompts in both the accounts of physicians and patients. Both, the web app and the Rhekiss-app contain error prompts for the patients.

Neither the physician nor the patient can look at each other's data to be able to collect intimate data without concern. Moreover, the data are only available to the data analysis team which does not have access to any personal data. The physicians can see all patients enrolled by them in the register. In addition, they are able to see the data entered by them as long as the questionnaire/form is not (auto-) submitted. The patients do not see which other patients have been enrolled or any data of other patients but all data entered by them until the form has been (auto-) submitted.

Positive ethics votes have been retrieved from the Charité University Medicine Berlin (EA1/0757/15) and the Medical Faculty Heinrich-Heine-University Duesseldorf (internal number 5114) and—where required—also from other ethical boards throughout Germany (available on request). In addition, data protection approval has been obtained. The register was registered to the German Clinical Trials Register (DRKS) with the identifier DRKS00024215 retrospectively on 6 June 2021.

Rhekiss was established and is maintained with financing from the DRFZ in Berlin and from the Rheumazentrum Rhein-Ruhr e.V.

For the current analysis, data from the Rhekiss register were used from the start of the register on 15 September 2015 to 30 September 2020. As the information on the device used was only implemented since 1 October 2018, the analysis on devices included only forms submitted from 1 October 2018 to 30 September 2020.

Each form can be sent only once, either *via* mobile device or *via* desktop. The source of information for the form retrieved (i.e., desktop or mobile device) is detected automatically from the Rhekiss web app. If the information on the device was not provided, this was always due to technical issues. Observations with missing device information were excluded from this analysis. Depending on the device used to submit the form, data were stratified into two groups, i.e., mobile and desktop users. Outcome parameters as a module in Rhekiss, diagnosis, age at filling out the questionnaire, educational level, breastfeeding, and severity of disease were compared for the two groups. To compare response rates before and after the implementation of the Rhekiss-app, the forms were separated into two groups: submitted until 31 July 2017 and after 1 August 2017.

Based on the applied usual classifications of the educational level of patients in Germany, patients were divided into the following two groups for the analyses: “secondary school level or less” and “university entrance diploma.” The latter was regarded as a “high educational level.”

To evaluate whether the app increases the number of exploitable self-reported patient data, the proxies “self-rated health status” and “Rheumatoid-arthritis-impact-of-disease questionnaire (RAID)” were selected because these parameters were to be observed at every time point for all patients. For comparability reasons, self-rated health status is assessed as in the national German database of the German Collaborative Arthritis Centres, and the RAID questionnaire is implemented as a validated PROM (12, 13).

Non-parametric Wilcoxon test, Kruskal–Wallis-test, and chi-square-test were used to compare the two groups. *P*-values < 0.05 were considered statistically significant. The statistical analysis was performed using SAS^©^ 9.4.

## Results

Physicians working in hospital outpatient clinics (*n* = 50) and 97 working in private practises are participating in the register.

**Figure 2:**
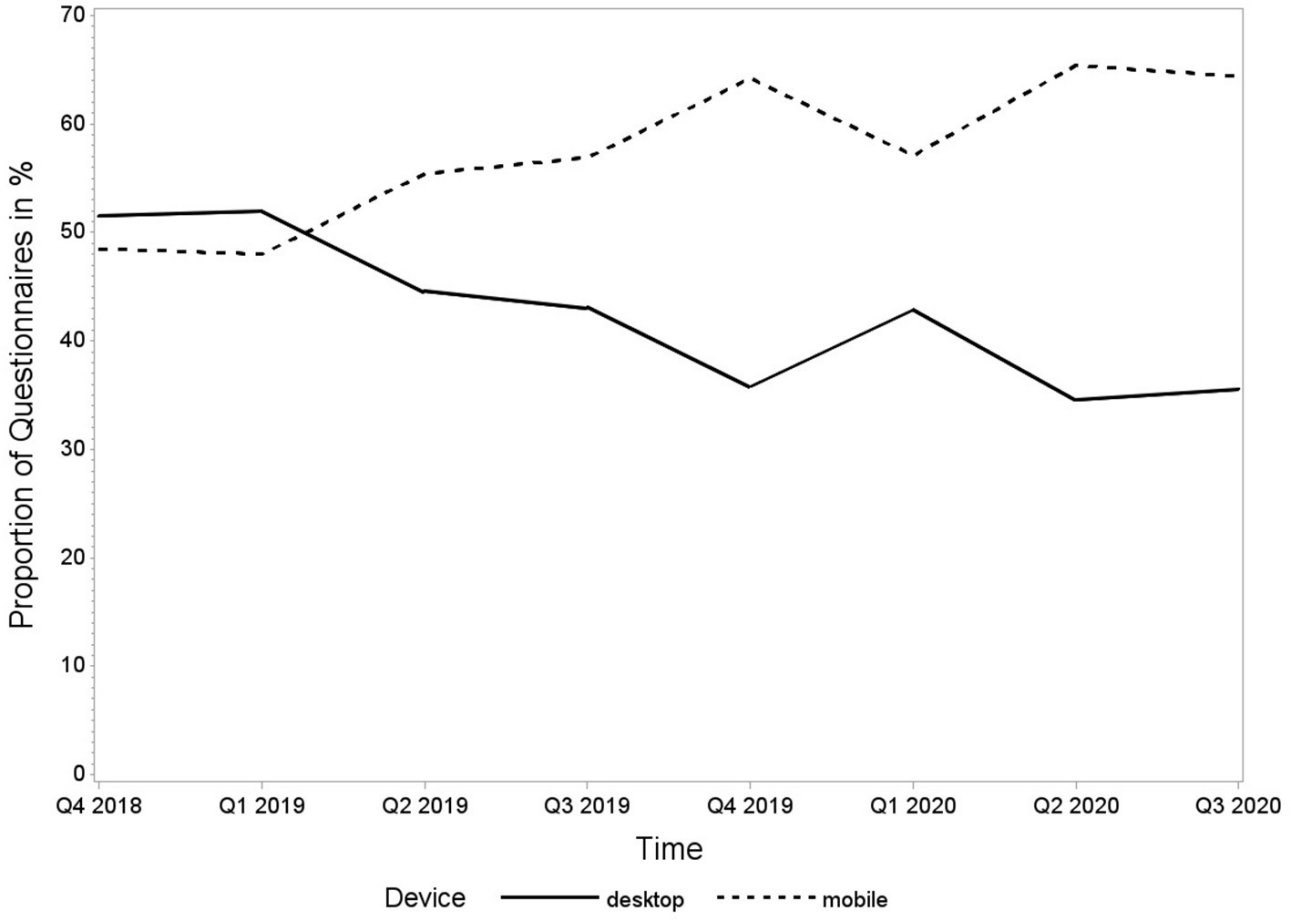
Proportion of questionnaires in percentage submitted by desktop and mobile devices from 1 October 2018 to 30 September 2020.

Until database closure for the analysis on 30 September 2020, 5,048 questionnaires had been submitted by 966 patients. Approximately half of the questionnaires received (2,380, 47%) were filled in *via* smartphone, 1,976 (39%) were sent from a desktop computer, 267 (5%) were filled in *via* tablet, and 12 (0.2%) *via* phablet. For 413 (8%) of the forms, the device was not detected due to technical issues. These observations were excluded from the analysis. The remaining 4,635 smartphone, tablet, and phablet observations were summarised into one mobile group for further analysis. The time course of desktop and mobile submitted forms over time is shown in Figure 2.

More than one questionnaire was filled in by 807 patients of which 434 (54%) used a mobile phone as the first device and 373 (46%) used a desktop computer. Some patients changed the device for their data reporting during their follow-ups in the register. The mode of data reporting changed in 24% of patients who started with desktop data entry and switched to mobile use and 12% who started with mobile and switched to a desktop for at least one questionnaire.

Distributions of forms among modules and diagnoses are shown in Table 1. About 15% of the questionnaires were submitted in the child wish module, 43% in the pregnancy module, and 42% in the post-partum module. In general, mobile forms were more frequently used than desktop forms. For psoriatic arthritis, more questionnaires were submitted by desktop (54%) and for all other diagnoses, mobile questionnaires were more common.

**Table 1:** Characteristics of patients who filled the forms either *via* desktop or mobile device.

	**Desktop**	**Mobile**	**Total**
**Questionnaires**, ***n*** **(%)**	1,976 (42.6)	2,659 (57.4)	4,635
**Module**			
Child wish *n* (%)	262 (37.2)	442 (62.8)	704
Pregnancy *n* (%)	836 (42.2)	1,147 (57.8)	1,983
Post-partum *n* (%)	878 (45.1)	1,070 (54.9)	1,948
**Diagnosis**			
Rheumatoid arthritis *n* (%)	557 (39.6)	849 (60.4)	1,406
Systemic lupus erythematosus *n* (%)	379 (45.9)	447 (54.1)	826
Other connective tissue diseases *n* (%)	335 (43.4)	437 (56.6)	772
Other Spondyloarthritis *n* (%)	277 (38.7)	438 (61.3)	715
Juvenile idiopathic arthritis *n* (%)	131 (43.2)	172 (56.8)	303
Psoriatic arthritis *n* (%)	146 (53.9)	125 (46.1)	271
Vasculitis *n* (%)	109 (49.1)	113 (50.9)	222
Autoinflammatory syndroms *n* (%)	42 (35.0)	78 (65.0)	120
**Age at questionnaire submit, mean (SD)**	34.2 (3.9)	33.1 (4.1)	33.6 (4.1)
**Age at questionnaire submit**, ***n*** **(%)**			
≤ 30 years	334 (32.1)	707 (67.9)	1,041
31–40 years	1,550 (45.6)	1,850 (54.4)	3,400
≥41 years	92 (47.4)	102 (52.6)	194
**Patients**, ***n*** **(%)**	412 (44.5)	514 (55.5)	926
**Number of questionnaires per patient, mean (SD)**	2.1 (3.1)	2.9 (3.4)	5 (3.5)
**Severity of disease (physician based)**, ***n*** **(%)**			
Asymptomatic	28 (8.4)	41 (9.7)	69
Mild	116 (34.8)	139 (32.9)	255
Moderate	145 (43.5)	187 (44.3)	332
Severe/very severe	44 (13.2)	55 (13.0)	99
**Self-reported education**, ***n*** **(%)**			
Secondary school level or less	70 (20.8)	140 (37.6)	210
University entrance diploma	266 (79.2)	232 (62.4)	498
Pregnancies, *n*	230 (43.6)	297 (56.4)	527
**Breast feeding**, ***n*** **(%)**			
Yes	157 (82.2)	211 (74.6)	368
No	34 (17.8)	72 (25.4)	106

The average age of desktop users was 34 years, while patients who completed their questionnaires by mobile devices were significantly younger with a mean age of 33 years (*p* = 0.0001). The group of patients under 30 years of age had the highest proportion of forms filled in by mobile devices (68%); in this age group, no difference of education level was detectable between the mobile and desktop users. Further details are listed in Table 1.

Out of 926 patients, mobile users submitted an average of 2.9 and desktop users of 2.1 questionnaires (*p* = 0.0001). The education level differed significantly between the two groups (*p* = 0.0001). Significantly more desktop users reported a high education level (79 vs. 62% in mobile users; *p* = 0.0001). In the group of desktop users, the proportion of breastfeeding patients was higher (82%) than in the mobile user group (75%), the difference was statistically not significant (*p* = 0.05). The distribution of severity of disease in desktop users did not differ statistically from mobile users (*p* = 0.05).

To compare the proportion of patient questionnaires sent by a specific device before and after the implementation of the Rhekiss-app, response rates were analysed for every time point. The proportion of submitted forms increased by 5% on average and detailed results for all time points are shown in Table 2.

**Table 2:** Proportion of patient reported forms received before and after the implementation of the Rhekiss-app (100% was assumed as the expected number of patient forms at the corresponding time point).

**Time point of the patient questionnaire**	**Patient questionnaire submitted**	**Before implementation of the app *n* (%)**	**After implementation of the app *n* (%)**	***p*-value**
First trimester	Yes	411 (75.1)	452 (77.0)	0.462
	No	136 (24.9)	210 (23.0)	
Second trimester	Yes	336 (77.6)	381 (78.7)	0.682
	No	97 (22.4)	166 (21.3)	
Third trimester	Yes	270 (73.2)	357 (73.3)	0.965
	No	99 (26.8)	208 (26.7)	
3 months post-partum	Yes	139 (56.0)	257 (60.0)	0.309
	No	109 (44.0)	289 (40.0)	
6 months post-partum	Yes	84 (56.0)	242 (64.7)	0.063
	No	66 (44.0)	226 (35.3)	
12 months post-partum	Yes	48 (44.9)	227 (57.2)	0.023
	No	59 (55.1)	271 (42.8)	

Regarding the completeness of data in the submitted forms, overall, the self-assessed health status was evaluable in 87% and RAID in 86% of the patients. For both scores, a significant difference between desktop and mobile was detected: forms filled-in on a desktop computer were more often completed but this was influenced by the different levels of education of desktop and mobile users (as shown in Table 3). Desktop users with university entrance diploma filled in the self-reported health status and the RAID more often complete than mobile users with university entrance diploma (72 vs. 57% and 72 vs. 56%, respectively). In patients with lower education levels, this was the opposite and the completeness was higher in mobile device users.

**Table 3:** Self-reported health status and rheumatoid-arthritis-impact-of-disease questionnaire (RAID) as proxies for the quality/completeness of patient reported outcome instruments filled in with differing devices.

	**Desktop *n* (%)**	**Mobile *n* (%)**	**Total *n* (%)**	***p*-value**
**Self-reported health status (one item)**
Complete	1,744 (88.3)	2,287 (86.0)	4,031 (87.0)	0.025
Not filled in	232 (11.7)	372 (14.0)	604 (13.0)	
**RAID (4 items)**
Complete	1,731 (87.6)	2,257 (84.9)	3,988 (86.0)	0.009
Filled in partially	1 (0.1)	9 (0.3)	10 (0.2)	
Not filled in	244 (12.3)	393 (14.8)	637 (13.7)	

## Discussion

Rhekiss is set-up as a web-based online pregnancy register for physicians and patients with IRDs. A GDPR conforms app for mobile devices provides an additional data entry option for patients. To our knowledge, this is the first study in rheumatology that evaluated an app as a tool for data documentation in a web-based register, thus, filling an important knowledge gap.

The Rhekiss-app was used for data acquisition from more than half (57%) of our patient questionnaires and its use remained high over the observed period, indicating good acceptance of the mobile documentation option. We anticipated this, as the opportunities and requirements of being spatially mobile are part of today's everyday life and normality of young people (14), and even though it has been reported that mobility in pregnant women is lower (15). Our data confirmed that mainly younger participants used the mobile app. It is in line with the findings that more than 95% of young people until the age of 39 years use smartphones, usually stick to their phone, and use it for internet access frequently (16).

Even though it could not be taken for granted that patients would follow our BYOD concept for self-reported data documentation, e.g., due to security concerns for data entry when installing or other, more technical aspects, such as compatibility issues, the app was accepted in all groups of our Rhekiss participants. We may have been benefited from the fact that the Rhekiss-app development was performed in the university/institutional, tertiary care context, and that it was not driven by commercial interests. Within a feasibility study of a patient diary set up as an app, the BYOD concept was accepted by patients with rheumatoid arthritis that were, however, older than the analysed Rhekiss cohort (17). In a similar approach, the register for “Breast cancer care for patients with metastatic disease” implemented an app for PRO collections (18). The authors were able to show that an app in a BYOD approach is feasible and accepted in incurable patients (18). Additionally, the BYOD approach has been recommended to ensure high acceptance of ePROMs of patients and the “conservation of instrument measurement equivalence” (19).

Socioeconomic status is assumed to influence the usage of the “new” technologies (20). In Rhekiss, more patients with higher education used the desktop for data entry. This may be due to the more regular availability of desktops (such as notebooks and laptops) in higher educated persons needing the device, e.g., for business reasons, and because desktops are prohibitively expensive for many other families (21). Even if we face more IT-savvy and well IT-equipped young patients of childbearing age, not every patient may fulfil the prerequisites to use apps (e.g., old operating system) (17) and, thus, need to rely on a desktop for data completion, e.g., with caring physicians, friends, or family members. The application modes showed a statistically significant difference in mean age, but we consider this difference of 1 year not relevant.

Prior to the Rhekiss-app development, we assumed that it will be particularly comfortable for breastfeeding women to enter information into an app with greater flexibility. However, our data did not confirm this assumption as breastfeeding mothers used the desktop more often. This finding may be driven by the fact that research with parents of older children suggests that engagement of parents with technological devices (e.g., mobile devices) in the presence of their children decreases the quality of parent-child interactions (22), which mothers may want to avoid in the early phase of life.

With the implemented app, response rates of questionnaires increased by 5% in the mean, being highest “12 months after post-partum.” Thus, we partially met one of our research goals. As missing data can badly affect the data quality, score calculations, and limit the usefulness of PROM (17), we investigated the number of evaluable register data retrieved from the different devices. Our two proxies used revealed that data entry *via* the desktop was significantly more often complete, suggesting that in a register desktop use should be preferred for data entry. However, the difference of the percentages is still small (~3%), raising the question of the clinical and health services research related meaningfulness of these results, which needs further clarification. According to our data, one contributing factor is given by the prevalent school education of patients, which cannot be controlled by the register. Perceptions and expectations towards the application might also depend on the underlying disease, requiring a user-centred design and implementation of a web-based register, similar to the recently reported e-register approach reported from Bogale et al., and other published recommendations for mobile health apps for patient self-management (23, 24).

Patient dashboards in which the entered data and also missing values of the different assessment periods (childbearing to postpartum) are displayed—e.g., also in comparison with results from other patients—could increase response and completeness rates in both modes of data acquisition. Caution must be taken when implementing them, as the dashboard could be seen as an intervention in the patient journey that would have to be evaluated and, moreover, could be restricted by the medical device regulation and other, e.g., country-specific, regulatory requirements (25–27). In Rhekiss, reminders for the questionnaire are sent out through the application only once when the corresponding module (e.g., first/second trimester) is activated. More advanced targeted client communication (e.g., more reminders) may be an option to increase patient participation, although the value of the targeted client communication approach *via* mobile devices has been questioned in a Cochrane review (28). Increased numbers of error prompts could be useful, considering that the option to leave questions unanswered is regarded as a relevant matter of choice (17, 29).

In the medical context, apps are typically used for quantified self-reported data, self-management programs (e.g., for maternal health behaviour and maternal and infant health), companion apps, or as apps for other interventions (e.g., nutrition and smoking cessation) (30–32). Although it has recently been reported that medical app use in patients with rheumatic disease in Germany is quite low, more than 97% of the patients conveyed that they would consent to share their mobile app data for research purposes (33). Thinking this through further, this could provide an opportunity to include this “new” type of data in the Rhekiss register to provide health services research with relevant information on mobility and physical activity during pregnancy in women with IRDs.

## Limitations

Our data represent data from Germany only; studies on the use of mobile devices in larger and international cohorts are warranted, especially when used in populations with very different characteristics, e.g., in more rural regions or regions with social or economic marginalisation. Our patients already showed a broad spectrum of disease severity, but more data from patients considering disease activity and/or assessments of functional limitations (that are, however, hardly validly assessable in pregnancies) are warranted as, e.g., active arthritis may limit the use of mobile devices. We do not have direct feedback from patients, e.g., regarding the highly relevant issue of user experience with our web-based system, which highlights the need for further elaborations to optimise our modern register app and, thus, to reduce potential technical barriers to ameliorate safety of mother and child. Since user experience (UX) is considered a relevant performance indicator of today's eHealth evaluations and has been applied in rheumatology (34, 35), one aspect of our research should also focus on UX evaluations of the Rhekiss-app. The implementation of these results could lead to user-relevant modifications (e.g., better graphical interfaces and display of forms).

It should be noted that our analyses look at the first implemented version of the app. Thus, based on the present data, we now explore options for further development of the Rhekiss-app with the technical partners to optimise data collection further. In addition, it may be useful to offer an online tutorial as a training opportunity for patients as due to legal obligations only caring physicians can get in touch with them directly.

## Conclusions

Apart from still ongoing hardware and software developments and other technological achievements, web-based apps for observational cohorts and/or future (joined) registries need to take requirements and developments for mobile devices into account. Especially young, mobile patients with IRD valued the opportunity of mobile entry of patient-reported data. Mobile data entry was preferred in most IRD diagnoses and independent of the module allocation. However, it needs to be considered that the mobile approach may have an impact on data quality and concepts for improving data quality should be implemented. It is, therefore, necessary to continue the incessant evaluation of digital apps, also with regard to the validity of the data collected. Rheumatology professionals, patients, and all other relevant stakeholders need to be engaged to effectively incorporate the new tools at the points of care and in the context of remote patient management, and in clinical register research.

## Data Availability Statement

The data supporting the conclusions of this article are available on reasonable request by the authors.

## Ethics Statement

The study involving human participants was reviewed and approved by the Ethics Committee of Charité University Medicine Berlin (EA1/0757/15, date 31st March 2015) and the Medical Faculty Heinrich Heine University Duesseldorf (internal number 5114). The patients provided their written informed consent to participate in the register.

## Author Contributions

CB, RF-B, JR, AS, MS, AW, and AZ designed, performed, analysed the study, drafted the manuscript, and analysed the data. All authors contributed to the manuscript, read and approved the submitted version, confirm being the sole contributors of this study, and have approved it for publication.

## Funding

Rhekiss was established and maintained with financing from the DRFZ in Berlin and from the Rheumazentrum Rhein-Ruhr e.V.

## Conflict of Interest

The authors declare that the research was conducted in the absence of any commercial or financial relationships that could be construed as a potential conflict of interest.

## Publisher's Note

All claims expressed in this article are solely those of the authors and do not necessarily represent those of their affiliated organizations, or those of the publisher, the editors and the reviewers. Any product that may be evaluated in this article, or claim that may be made by its manufacturer, is not guaranteed or endorsed by the publisher.
